# Fetor of Breath

**Published:** 1902-02

**Authors:** 


FETOR OF BREATH.

| | | |
|---|---|---|
| ℞ | Thymoli . . . . . . . . . . . . . . . . . . . . . . . | gr. viij |
| | Alcohol . . . . . . . . . . . . . . . . . . . . . . . | fl. ℥ j |
| | Glycerini . . . . . . . . . . . . . . . . . . . . . . | fl. ʒ iv |
| | Formaldehydi (40 per cent. sol.) . . . . . . . . . . . | gtt. viij |
| | Aqua, q. s. ad . . . . . . . . . . . . . . . . . . . . | fl. ℥ iij |

M. Sig.—Use as a mouth wash. Indication: Used in fetor from decayed teeth.—*Maryland Med. Jour.*

